# Induction of Autophagy and Apoptosis via PI3K/AKT/TOR Pathways by Azadirachtin A in *Spodoptera litura* Cells

**DOI:** 10.1038/srep35482

**Published:** 2016-10-18

**Authors:** Xuehua Shao, Duo Lai, Ling Zhang, Hanhong Xu

**Affiliations:** 1 State Key Laboratory for Conservation and Utilization of Subtropical Agro-bioresources, Key Laboratory of Natural Pesticide and Chemical Biology of the Ministry of Education, South China Agricultural University, Guangzhou 510642, China; 2 Institute of Fruit Tree Research, Guangdong Academy of Agricultural Sciences, Guangzhou 510640, China; 3 College of Life Science and Technology, Jinan University, Guangzhou 510642, China

## Abstract

Azadirachtin is one of the most effective botanical insecticides and has been widely used in pest control. Toxicological reports show that azadirachtin can induce apoptosis in various insect cell lines. However, studies of azadirachtin-induced autophagy in cultured insect cells are lacking. This study reports that azadirachtin A significantly inhibits cell proliferation by inducing autophagic and apoptotic cell death in *Spodoptera litura* cultured cell line (SL-1 cell). Characteristic autophagolysosome and Atg8-PE (phosphatidylethanolamine) accumulation were observed by electron microscopy and western blotting, indicating that azadirachtin triggered autophagy in SL-1 cell. Furthermore, azadirachtin inhibited survival signaling by blocking the activation of PI3K, AKT and the down-stream target of rapamycin. Similar to the positive control of starvation, azadirachtin induced the activation of insulin receptor (InR) via a cellular feedback mechanism. In addition, the autophagy-related 5 (Atg5), a molecular switch of autophagy and apoptosis, was truncated (tAtg5) to trigger cytochrome c release into the cytoplasm under azadirachtin stress, which indicated that azadirachtin induced apoptosis through autophagy. Our findings suggest that azadirachtin primarily induced autophagy in SL-1 cell by dysregulating InR- and PI3K/AKT/TOR pathways, then stimulated apoptosis by activating tAtg5.

Autophagy is a lysosome-mediated process of cellular self-destruction, in which the cytoplasmic cargos (such as aged proteins, misfolded proteins or damaged organelles) are sequestered in double- or multi-membrane vesicles (autophagosomes) and delivered to lysosomes for bulk degradation^1^,^2^,^3^,^4^. Under normal conditions, autophagy occurs at a low basal level in cells to maintain homeostasis. However, in unfavorable conditions such as nutrient deprivation, oxidative stress or infection, autophagic cell death will occur. The most prominent feature of autophagy is the formation of a double-membrane sequestering compartment. This transient organelle surrounds part of the cytoplasm and matures into an autophagosome, which subsequently fuses with the lysosome to allow degradation of the cargo^2^. Autophagy is governed by a series of *Atg* genes. During this process, Atg8 (LC3, microtubule associated protein 1-light chain 3) is specifically cleaved and lipidated to become LC3-II. LC3-II, which is recruited to the autophagosome membrane. Increased levels of LC3-II proteins and LC3-II-containing autophagosomes are important biomarkers of autophagy^5^. Autophagy is considered a type II programmed cell death (PCD), with the hallmark of accumulated autophagosomes in dying cells^6^,^7^,^8^. Apoptosis, a type I form of PCD, is executed by activated caspases, which are a family of cysteine proteases that participate in signaling cascades. Apoptosis culminates in cellular shrinkage with nuclear chromatin condensation, nuclear fragmentation, formation of apoptotic bodies and eventual phagocytosis^9^. Autophagy promotes cell survival against apoptosis, but extensive autophagy may also cause cell death in certain circumstances^10^. Although significant advances have recently been made regarding the functional relationship between autophagy and apoptosis, very little is understood about the regulation of their crosstalk under cellular stress.

There are many triggers in autophagy induction, including starvation, irradiation, chemicals, viral infection, and cell stress^11^,^12^,^13^. Recently, multiple signaling pathways have been found to be involved in the modulation of autophagy. The target of rapamycin (TOR), a downstream component of the phosphatidylinositol 3-kinase (PI3K)/AKT pathway, is critical for autophagy. TOR is an evolutionarily conserved kinas that maintains cell growth and survival^14^,^15^,^16^. Generally, the upstream signal PI3K/AKT or nutrients including insulin activate TOR thus suppressing autophagy^11^. Insulin-mediated activation of PI3K serves as a second messenger and recruits AKT to the plasma membrane^17^. Once properly localized to the membrane, AKT becomes activated by phosphorylation and in turn phosphorylates a number of downstream targets that ultimately regulate cell growth. AKT also stimulates protein synthesis through TOR activation. However, as a sensor of nutrient status, TOR is suppressed in the absence of growth factors thus activating autophagy^11^.

Azadirachtin A (AZA) is an effective botanical insecticide that is primarily isolated from the seed kernel of the neem tree *Azadirachta indica* A. Juss (Meliaceae)^18^,^19^,^20^ and has been widely used as an alternative to synthetic pesticides for controlling various types of agricultural pests because of its non-persistence in the environment and low toxicity against non-target beneficial organisms^21^,^22^,^23^. AZA has potent antifeedant and growth inhibitory activities against insects. Moreover, AZA can affect cell proliferation and induce apoptosis in various insect cell lines^24^,^25^,^26^,^27^. Previous studies have shown that AZA provoked potent growth-inhibitory effects in *Drosophila* larvae by dysregulating the insulin/insulin-like growth factor signaling pathway^28^ and inducing programmed death, cytoskeletal damage and autophagic vacuoles on *Trichoplusia ni* BTI-Tn-5B1-4 cells by disrupting lysosomal function^29^. Recently, AZA-induced apoptosis has been shown to involve lysosomal membrane permeabilization and cathepsin L release in *S. frugiperda* Sf-9 cells^30^. However, given the lack of studies on the autophagy/apoptosis signaling pathway involved in AZA-induced proliferation arrest in cultured insect cells, the mechanism of AZA-induced autophagy remains largely unknown. In the present study, we discovered that AZA-induced TOR-dependent autophagy and autophagic cell death in SL-1 cells, and a crosstalk occurred between AZA-induced autophagy and caspase activity when tAtg5 was activated.

## Results

### Effects of AZA on autophagy and proliferation in SL-1 cells

**Figure 1:**
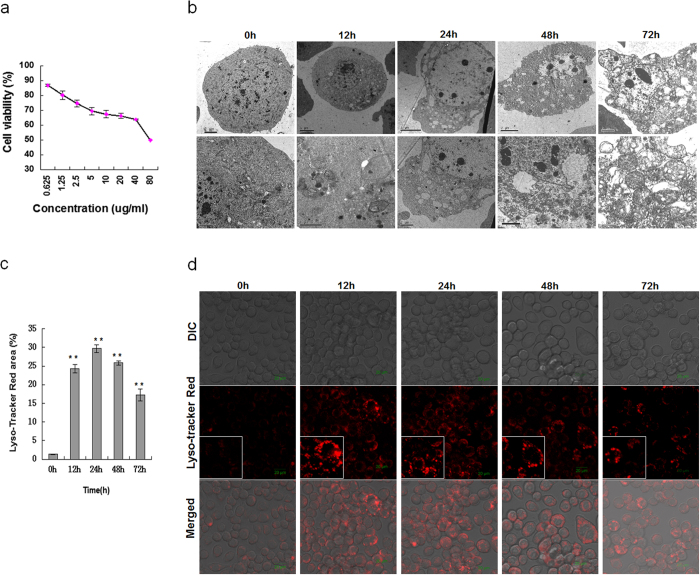
Analysis of autophagy and proliferation in SL-1 cells treated with AZA. (**a**) Effects of AZA on cell proliferation. (**b**) Representative photographs of the ultrastructure of cells treated with or without AZA, obtained by TEM. (**c**) Percentage of autolysosome. Data are the mean ± S.D. **p < 0.01 compared with 0 h. (**d**) Lysosomes detected by Lyso-Tracker Red staining intensity with AZA treatment.

Previously, we successfully demonstrated that AZA induced programmed death and autophagic vacuoles in *Trichoplusia ni* BTI-Tn-5B1-4 cells^29^, but the mechanism underlying AZA-induced autophagy is still obscure. In the present study, we first evaluated AZA cytotoxicity using the WST-8 test to verify whether AZA was capable of inducing autophagy in SL-1 cells. The results showed that SL-1 cell proliferation was inhibited in a dose-dependent manner after 24 h exposure. The inhibition activity can be significantly observed at a low concentration of 0.625 μg/mL. The cell viability rates were 80.19%, 74.72%, 69.41%, 67.43%, 66.22%, 63.83% and 49.84% at the concentrations of 1.25, 2.5, 5, 10, 20, 40 and 80 μg/mL, respectively (Fig. 1a).

We then determined the effects of AZA on autophagy in SL-1 cells using transmission electron microscopy (TEM), LysoTracker Red and GFP-LC3 analyses three effective indicators of autophagy^16^,^31^,^32^. As monitored using TEM, the initial cells without AZA treatment had an integrated cell nucleus, evenly distributed chromatin, and discrete organelles. However, the cell was seriously damaged with autolysosome formation after AZA treatment. At the 12 h time point, SL-1 cells treated with AZA exhibited many membrane-bound vesicles containing organelles and cellular fragments, and double-membrane autophagolysosomes were clearly observed. Put 24 h and 48 h in parenthesis, the vacuoles steadily intensified in the cytoplasm, the nucleus diminished and chromatin accumulated; the cell eventually collapsed at 72 h (Fig. 1b). Similarly, LysoTracker Red staining intensity increased in the AZA-treated cells (Fig. 1d). The LysoTracker-labelled lysosome spots consisted of only 1% of the cytoplasm area at 0 h (without AZA), but sharply increased to approximately 24% at 12 h after AZA exposure. The LysoTracker Red staining intensity reached the maximum of approximately 30% after 24 h exposure then declined at 48 and 72 h but still exceeded 16% (Fig. 1c).

**Figure 2:**
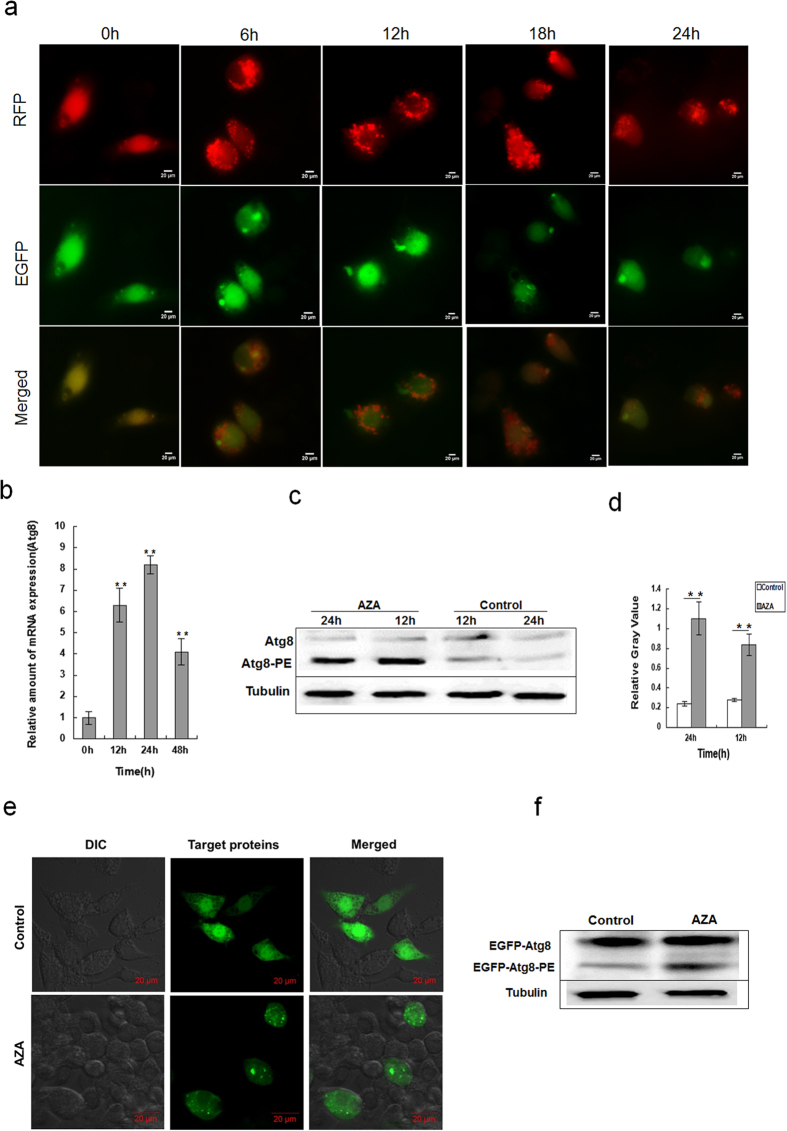
The expression level of Atg8-PE and subcellular distributions in SL-1 cells. (**a**) EGFP and RFP puncta represent autophagic vacuoles and display differential pH sensitivity. (**b**) Cells were induced by AZA, and the relative expression of ATG8 mRNA in different treatments was determined by qRT-PCR. Data are the mean ± S.D. **p < 0.01 compared with 0 h. (**c**) Western blot of Atg8-PE level induced by AZA in SL-1 cells. Control was without treatment, tubulin was used as a loading control for the western blot analysis. (**d**) The quantification of Atg8-PE from 3 experiments performed in triplicate. Data are the mean ± SD. **p < 0.01 compared with the control group. (**e**) pIEX-4-EGFP-Atg8 transfected into SL-1 cells, with AZA treaments for 12 h, and the puncta of EGFP-Atg8-PE conjuncted with the autophagosome. (**f**) Western blot of EGFP-Atg8-PE by EGFP antibody from EGFP-Atg8-PE over-expressed cells induced by AZA. Control was without treatment.

In addition, AZA-induced autophagy was further confirmed using RFP-GFP tandem fluorescent-tagged LC3; this construct is a fusion of LC3 and red (RFP) and green (GFP) fluorescence proteins and is one of the most widely used markers for detecting autophagosomes^33^. We used RFP-GFP-LC3 to monitor the autophagy process of autophagosome-lysosome fusion and autophagic flux based on the different pH stabilities of GFP and RFP^34^, as the acidic environment of the lysosome quenches the GFP signal but not the RFP signal. The yellow merged image represents the autophagosomes, whereas the merged images with red puncta indicate autophagic flux with autolysosome formation. As observed under a fluorescence microscopye (Fig. 2a), the red LC3 puncta (RFP) significantly increased compared with the control (0 h). However the percentage of cells with RFP-GFP colocalization decreased because of the autophagosome clearance. This finding indicates that AZA promoted the fusion of autophagosomes and lysosomes, thereby facilitating maturation of autophagosomes into autolysosomes. These results conclusively demonstrated that AZA-induced autophagy in SL-1 cells by increasing autophagosome-lysosome fusion.

### AZA-induced Atg8-PE expression level in SL-1 cells

The autophagy-related gene *Atg8* is highly conserved among insects. qRT-PCR analysis showed that the expression levels of the *Atg8* gene significantly increased after AZA exposure for different times, and the highest expression was detected at 24 h (Fig. 2b), indicating that *Atg8* was regulated at the transcriptional level by AZA. We analyzed the expression and processing of the ATG8 protein, a factor involved in the expansion of the isolation membrane that leads to autophagosome formation^2^, to confirm the gene expression results. When presented with unfavourable conditions, such as nutritional deprivation and chemical stimulation, the ATG8 protein is primarily conjugated to the lipid PE. We performed western blot analysis with anti-Atg8 antibody on protein extracts from AZA-treated cells (Fig. 2c). Bands corresponding to the PE-conjugated form of Atg8 (Atg8-PE), a marker associated with completed autophagosomes^35^, were detected at 12 and 24 h. Their expression levels were more than 4-fold that of the control (Fig. 2d). Moreover, induction of Atg8-PE conjugation bound to the autophagic membrane by AZA was assessed through fluorescence analysis using EGFP-Atg8 as a marker exhibiting the same behavio as Atg8. As shown in Fig. 2e, the bright fluorescent spots were distinctly detected in cells exposed to AZA for 12 h but not in the control cells that were only expressing EGFP-Atg8 scattered green fluorescence, which was further demonstrated by an increase in EGFP-Atg8-PE puncta formation from the same transfected cells (Fig. 2f). Taken together, these findings further confirmed AZA-induced autophagy in SL-1 cells at the transcriptional and translational levels.

### AZA-induced autophagy by inhibiting the PI3K/AKT/TOR pathway

**Figure 3:**
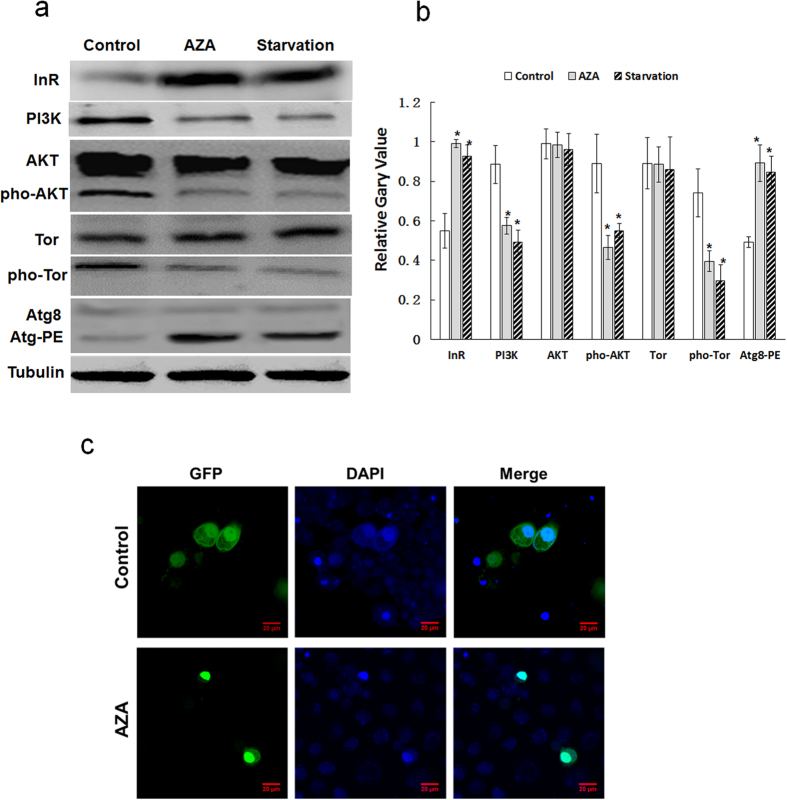
Effects of AZA and starvation treatment on the InR-PI3K-AKT signaling cascade, FOXO and ATG8 in SL-1 cells. (**a**) Cells were treated with AZA (2.5 μg/mL) and PBS for 24 h, control was without treatment, tubulin was used as the internal control. (**b**) The quantitative results from target proteins of three independent experiments. Data are the mean ± S.D. *p < 0.05 compared with control group. (**c**) pIEX-4-FOXO-GFP transfected into SL-1 cells were treated with AZA for 24 h and stained with DAPI.

The PI3K/AKT/TOR signaling pathway was investigated in SL-1 cells to explore the molecular mechanism underlying the autophagy induced by AZA. The PI3K signaling pathway negatively controls autophagy. The results demonstrated decreased PI3K (85KD) in SL-1 cells following treatment with 2.5 μg/mL AZA for 24 h (Fig. 3a). We thus investigated whether AZA-induced autophagy occurred through TOR inhibition because TOR acts as a well-conserved and central regulator in autophagy induction-and AKT modulates TOR activation^36^,^37^. The levels of phospho-AKT (p-AKT) and phospho-TOR (p-TOR), both of which are TOR substrates, in the AZA-treated SL-1 cell lines were measured. As shown in Fig. 3a,b, AZA significantly inhibited the phosphorylation levels of AKT and TOR, but it did not significantly affect the expression levels of the total forms of AKT and TOR. As expected, a significant increase in the formation of AZA-induced Atg-PE conjugation was also observed in SL-1 cells. Then, we examined whether AZA had any effects on the nutrition signaling pathway. The results showed that AZA increased the expression of insulin receptor (InR) (Fig. 3a,b), a regulator of cell growth and proliferation, indicating AZA played a feedback role by activating downstream regulatory factors in nutrient-mediated signaling pathways. To test whether InR was feedback-regulated by downstream activated-FOXO, the overexpression vector pIEX-4-FOXO-GFP was transfected into SL-1 cells. The results showed that AZA could cause the translocation of FOXO from the cytoplasm to the nucleus and activate transcription activity (Fig. 3c). These experiments demonstrated that AZA-induced autophagy in SL-1 cells was via dysregulating the InR-PI3K/AKT/TOR signalling pathway.

### AZA-induced apoptosis in SL-1 cells

**Figure 4:**
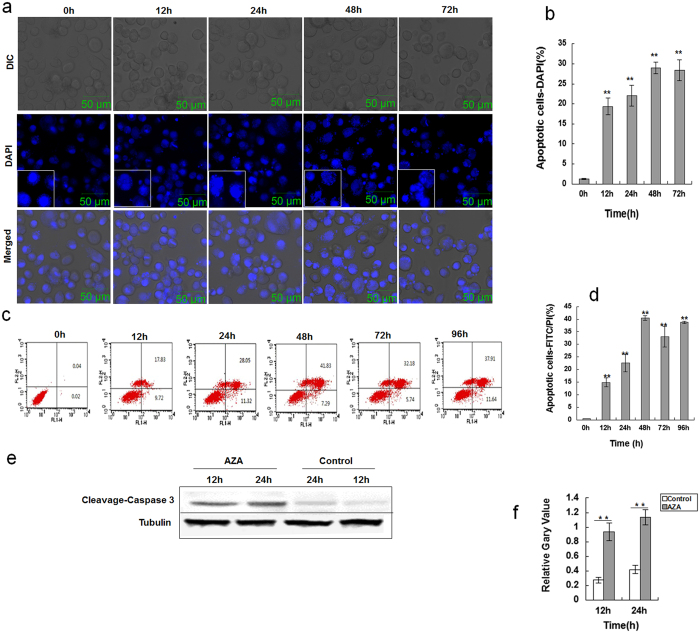
AZA- induced apoptosis in SL-1 cells. (**a**) SL-1 cells were induced for 0 h, 12 h, 24 h, 48 h or 72 h, following staining with DAPI for 10 mins. (**b**) Statistical table shows the quantification of DAPI staining intensity. Data are the mean ± S.D. **p < 0.01 compared with 0 h. (**c**) Evaluation of apoptosis of SL-1 cells using annexin V/PI staining and flow cytometry. All these cells were treated with 2.5 μg/mL AZA for various amounts of time. Cells were double stained with annexin-V-FITC and propidium iodide (PI) and analyzed by flow cytometry. Annexin-V-, PI- cells are live cells; annexin-V+, PI- cells are early apoptotic cells and annexin-V+, PI+ cells are late apoptotic or necrotic cells. (**d**) Flow cytometric analysis result. Data are the mean ± S.D. **p < 0.01 compared with 0 h. (**e**) Western blot of cleaved-caspase-3 level induced by AZA from 12 h and 24 h in SL-1 cells. Control was without treatment. Tubulin was used as a loading control for the western blot analysis. (**f**) The quantification of cleaved-caspase-3 from 3 independent experiments. Data are the mean ± S.D. **p < 0.01 compared with the control group.

Cellular morphological changes were observed by LSCM to evaluate the effects of AZA on apoptosis. As shown in Fig. 4a, the apoptotic bodies and floating cells gradually increased with the extension of induction time after AZA treatment. Analysis of nucleus morphology by DAPI staining intensity showed that the control group maintained intact and circular nuclei, whereas approximately 18–28% of the AZA-treated cells exhibited anomalous and swelled nuclei (Fig. 4a,b). Furthermore, the apoptotic cells were measured using flow cytometric analysis with annexin V-FITC and propidium iodide staining. The results indicated that AZA-induced apoptosis in SL-1 cells in a time-dependent manner. The apoptosis rates were dramatically enhanced after AZA exposure for 24 h (17.83%) compared with the control, and the maximum apoptosis rate of up to 41.83% occurred at 48 h (Fig. 4c,d). We further evaluated SL-1 cells using antibodies against caspase-3 as markers for apoptosis because cleaved caspase-3 is a critical executioner of apoptosis. Western blots showed that the expression levels of cleaved caspase-3 were significantly enhanced after AZA exposure for 12 and 24 h (Fig. 4e,f). These data conclusively indicated that AZA-induced apoptosis was a caspase-dependent type I process by activating the cleavage of caspase-3.

### Relationship between autophagy and apoptosis induced by AZA

**Figure 5:**
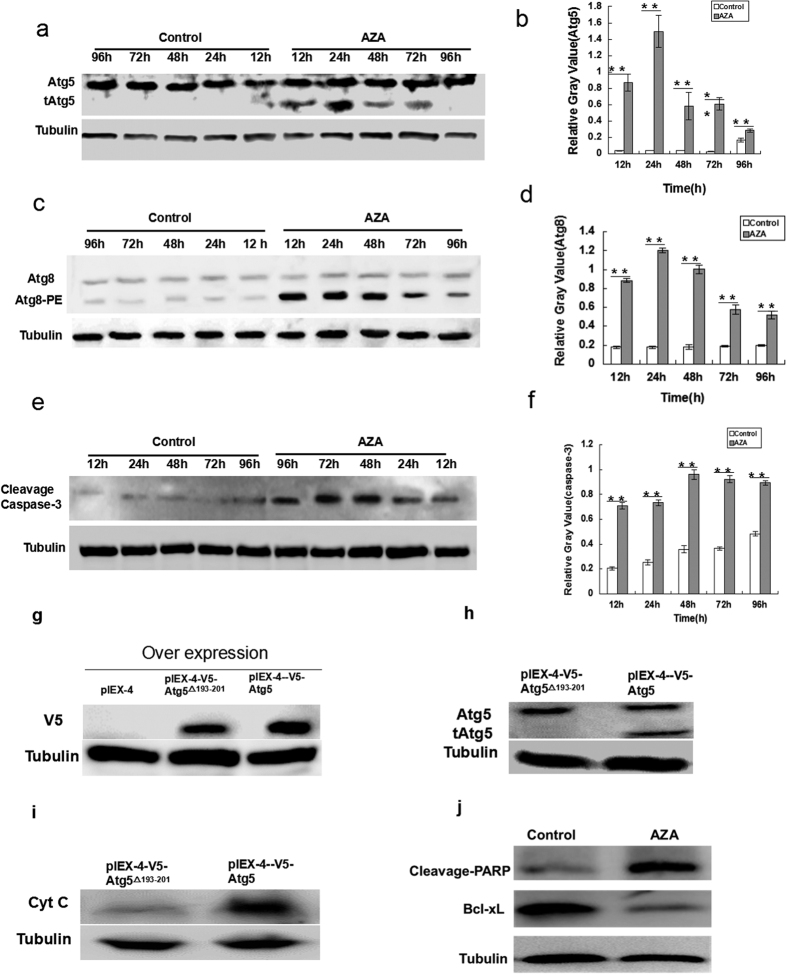
tATG5 plays key roles in autophagy preceding apoptosis in SL-1 cells. (**a,c,e**) Western blotting of ATG5, ATG8, and Caspase-3 induced by AZA for different times in SL-1 cells. The control group was without AZA treatment. Tubulin was used as a control for the western blot analysis. (**b,d,f**) The quantification of the target protein from 3 independent experiments. Data are the mean ± S.D. **p < 0.01 compared with control group. (**g–i**) Protein levels of V5, tAtg5 and Cyt C after V5-Atg5 or V5-Atg5^△193–201^ transfection into SL-1 cells followed by AZA treatment for 24 h. (**j**) Protein levels of Bcl-xL and cleaved-PARP by AZA treatment for 24 h.

Considering the important roles of autophagy and caspases in cell fate, we investigated whether crosstalk occurred between AZA-induced autophagy and apoptosis in SL-1 cells. We extracted total protein from the SL-1 cells treated with AZA for 12, 24, 48, 72 or 96 h. The expression levels of Atg8-PE, cleavage-caspase-3 and Atg5 were measured using western blots. The results showed an increase in Atg8-PE in a time-dependent manner following AZA treatment (Fig. 5c), accompanied by increases in cleavage-caspase-3 and Atg5 (Fig. 5a,e). In detail, the Atg8-PE and truncated Atg5 (24KD and tAtg5) expression levels simultaneously peaked after 24 h of AZA exposure, but cleavage-caspase-3 was still at the priming state (Fig. 5b,d,f). The highest cleavage-caspase-3 expression level was at 48 h. This result implies that there is a correlation between AZA-induced autophagy and caspase activity. To reveal whether tAtg5 could induce apoptosis, overexpression vectors pIEX-4-V5-Atg5 and pIEX-4-V5-Atg5^△193–201^ were transfected into SL-1 cells. The expression levels of V5 were measured by western blotting, the results showed that the wild-type Atg5 and mutant Atg5^△193–201^ proteins with V5-tag were both expressed in SL-1 cells (Fig. 5g). After treatment with AZA for 24 h, we found that overexpression of Atg5 could produce tAtg5, whereas Atg5^△193–201^ could not (Fig. 5h). Meanwhile, the expression levels of cytochrome c (CytC), as an apoptosis marker, in the cytoplasm were also measured; the western blot result indicated that CytC in the overexpressed Atg5 cells treated with AZA was significantly observed, whereas it was barely detected in the overexpression Atg5^△193–201^ cells (Fig. 5i). Correspondingly, the expression levels of the apoptosis inhibitor Bcl-xL were significantly reduced and the cleavage of PARP was activated (Fig. 5j). These data indicated that AZA-induced tAtg5 could promote CytC release to cytoplasm and cause apoptosis. Therefore, we speculated that induction of extensive autophagy by AZA triggered apoptosis in SL-1 cells.

## Discussion

Azadirachtin-induced cell cycle arrest and apoptosis in insect cells have been specifically reported in many studies^25^,^26^,^27^,^29^,^38^,^39^, and substantial research has been undertaken to explore the molecular mechanisms underlying the action of its cell proliferation inhibition. In previous studies, mitochondrial involvement and the up-regulation of p53 protein was observed in apoptosis induction when the cultured insect cells were treated with AZA^38^,^39^. AZA-induced apoptosis involved lysosomal membrane permeabilization and the release of lysosomal enzymes in *S. frugiperda* Sf9 cells^31^. A previous work had supported that azadirachtin- induced apoptosis and autophagic vacuoles in BTI-Tn-5B1-4 cells^29^. Srivastava *et al*. provided insight into mechanisms of apoptosis and autophagy by neem limonoids, and demonstrated that neem induces caspase-dependent and AIF-mediated apoptosis and autophagy in cancer cells^40^. In the present study, we provided the first confirmation that azadirachtin primarily induced autophagy in SL-1 cells by PI3K/AKT/TOR pathways at the molecular level and then stimulated apoptosis by activating tAtg5.

Autophagy has been known to promote cellular survival during nutrient depletion and to participate in a number of cellular and developmental processes, including cell growth control and programmed cell death^41^,^42^,^43^. In the absence of nutrients or under cell stress, damaged cytoplasmic organelles and proteins are degraded through an autophagy pathway to maintain cellular homeostasis and support self-survival. However, a high level of cell autophagy and apoptosis results in cell death^7^,^10^. In this present study, AZA was found to induce autophagic cell death in SL-1 cells. The distinct morphological markers autophagic vacuoles, autophagosomes and lysosomes were observed in the cytoplasm of AZA-treated cells after 12 h through TEM and Lyso-tracker Red staining. As one of the three principal methods of assessment, the membrane-associated form of LC3 (LC3 puncta) is widely used to monitor the number of autophagosomes^44^. In addition to LC3, Atg8 can also be used as a protein marker, which is primarily conjugated to the lipid PE, and increased Atg8-PE levels are associated with autophagy activation^35^. In the present study, the red number of LC3 puncta significantly increased after 12 h of AZA exposure, whereas the green LC3 puncta decreased (Fig. 2a). AZA elicited a distinct increase (more than 4-fold) in the expression level of the PE-conjugated form of Atg8 (Atg8-PE) (Fig. 2d). These results were also verified using fluorescence analysis (Fig. 2e).

Growth and nutrient intake are functionally linked processes during insect development and are regulated by the insulin/insulin-like growth factor signaling (IIS) pathway^45^,^46^,^47^. A previous study has shown that AZA inhibited growth and development by dysregulating the IIS pathway^29^. In the present study, we confirmed that the InR expression level was enhanced by AZA, similar to starvation treatment, in SL-1 cells (Fig. 3a). The InR signaling pathway is largely conserved in metazoans and is required for normal growth and development in insects. InR activates a cascade of events that leads to the phosphorylation of several adaptor proteins, including the insulin receptor substrates (IRSs). IRSs are linked to the activation of two main signaling pathways: the phosphatidylinositol 3-kinase (PI3K)/AKT pathway, which is responsible for most of the metabolic actions of insulin, and the Ras-mitogen activated protein kinase (MAPK) pathway that cooperates with the PI3K pathway to regulate cell growth and proliferation. The insulin-like growth factor (IGF) generally activates TOR to suppress autophagy and promote cell survival via the PI3K/AKT signaling pathway^11^,^48^. However, some PI3K inhibitors, such as LY294002 and wortmannin, induce autophagy by inhibiting the PI3K/AKT/TOR pathway^49^,^50^. Inhibition of the PI3K/AKT/TOR pathway is correlated with triggering autophagy in cancer cells^51^,^52^. Plumbagin, a natural naphthoquinone, induces autophagy by inhibiting the AKT-TOR pathway in A549 cells^53^. The present study demonstrated that AZA suppresses the expression levels of total PI3K protein. Moreover, AZA disrupted the TOR signaling pathway by inhibiting its phosphorylation, which is essential for TOR activity. Inhibition of TOR phosphorylation by AZA notably depended on inactivating phosphorylated AKT at Ser 505 (the activated form). These results indicated that AZA elicited autophagy in SL-1 cells via the PI3K-AKT-TOR pathway. Using PBS starvation as a positive control, AZA elicited the same effects as in nutrition deprivation. Because the dephosphorylation of AKT could activate the regulator FOXO, and the activated form of foxo feedback regulated and increased the expression of InR^54^. Indeed AZA caused the translocation of FOXO from the cytoplasm to the nucleus and activated the transcriptional activity (Fig. 3c). Based on the above, we proposed that AZA hindered the combination of insulin and insulin receptor, leaded to phosphorylation levels of InR changes and affected the phosphorylation levels of downstream signaling. However, we could not detect the phosphorylation levels of InR, because there is not specific antibody of p-InR in insect. The dephosphorylation of AKT activate the regulator FOXO and then induced the activation of InR via a cellular feedback mechanism under azadirachtin stress. It needs subsequent work to reveal how azadirachtin acts on the phosphorylation levels of InR.

Consistent with previous studies, we also observed that AZA had a pronounced effect on apoptosis in SL-1 cells. A question arose concerning how AZA- integrated autophagy and apoptosis inhibit cell proliferation. Therefore, we measured the protein expression levels, which are essential for autophagy and apoptosis in a time-dependent manner on SL-1 cells. The peak expression of autophagy-related protein Atg8 presented 24 h after AZA treatment (Fig. 5d). However, the peak expression of apoptosis protein was at 48 h (Fig. 5f). That is, peak expression of autophagy-related proteins precedes that of apoptosis. Autophagy and apoptosis are two distinct self-destructive processes that play essential roles in determining cell fate. Autophagy serves a pro-survival mechanism by providing sources of energy and biosynthetic building blocks during starvation. However, upon sustained stress conditions, cell death eventually takes place either by excessive autophagy or by the induction of apoptosis and/or necrosis pathways^55^. Atg5 has a pivotal role in autophagosome formation; it acts a molecular switch to regulate autophagy and apoptosis. Atg5 (31KD) can promote cell autophagy, whereas truncated Atg5 (24KD and tAtg5) promotes apoptosis^56^.

**Figure 6:**
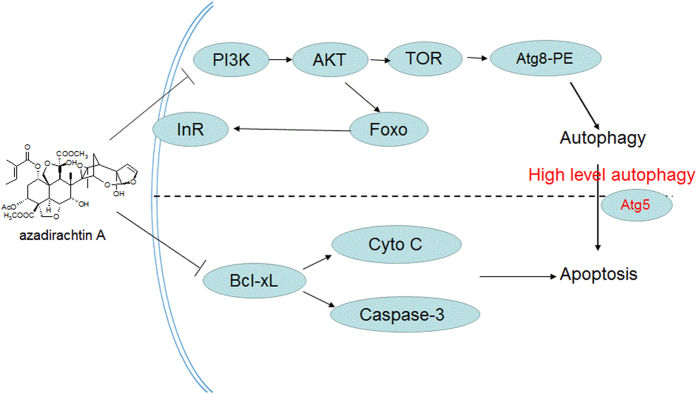
A proposed model of the molecular mechanism underlying azadirachtin-induced autophagy and apoptosis.

Atg5 is a key protein of autophagic cell death. Autophagosome formation involves a ubiquitin-like conjugation system in which Atg12 is covalently bound to Atg5 and targeted to autophagosome vesicles^57^. This conjugation reaction is mediated by the ubiquitin E1-like enzyme Atg7 and the E2-like enzyme Atg10^58^,^59^. Recent studies have shown that Atg5 (24KD) cleavage by calpain binding Bcl-xL induced apoptosis and Atg5 (33KD) induced autophagy^60^,^61^. Therefore, the Atg5 protein plays an important role in cell death and is a key factor between autophagy and apoptosis. In the present study, the expression of truncated Atg5 (24KD) was significantly increased after AZA treatment. Peak expression of autophagy-related proteins precedes that of apoptosis, which implies that the t-Atg5 protein is a key factor in autophagy and apoptosis in AZA-treated SL-1 cells. We found that overexpression of Atg5 could release cytochrome c into the cytoplasm, whereas overexpression of mutant Atg5^△193–201^ could not (Fig. 5h,i). Therefore, it can be observed that Atg5, as a molecular switch, initiated the transformation of autophagy to apoptosis after AZA treatment. To our knowledge, this is the first study to reveal a correlation between autophagy and apoptosis induced by AZA. Based on the analyses above, the molecular mechanism of autophagy and apoptosis induced by azadirachtin is summarized in Fig. 6.

In summary, we revealed that azadirachtin induced autophagic and apoptotic cell death in *S. litura* cells through the InR- and PI3K/AKT/TOR-mediated pathways. Azadirachtin is also involved in activating tAtg5 (Fig. 6). Thus, our data provide information on the mechanisms by which azadirachtin induces autophagy and apoptosis. However, further studies are needed to elucidate the mechanisms underlying the AZA-induced regulation of the nutrient-mediated signaling pathways.

## Methods

### Reagents, plasmid, and antibodies

Azadirachtin A (purity 95%) obtained from our laboratory was dissolved in DMSO and stored at −20 °C. The pIEX-4-GFP-RFP-LC3, pIEX-4-FOXO-GFP, pIEX-4–V5-Atg5 and pIEX-4-V5-Atg5^△193–201^ (inactivated deletion mutations in the calpain cleavage sites) plasmids and antibodies against Atg5 and Atg8 were provided by Prof. Y. Cao (South China Agricultural University). Monoclonal antibodies against caspase-3 (#9661S), PI3K (#4257), cytochrome c (#4272), V5-Tag (#13202), p-TOR (Ser2448, #5536), AKT(Ser505, #4054S), and Bcl-xL (#2764S) were purchased from Cell Signaling Technology, Inc. (Danvers, MA, USA). InR (sc-135949) was purchased from Santa Cruz Biotechnology, Inc. (Texas, USA). Anti-cleaved PARP antibodies [E51] (ab32064) were purchased from Abcam Trading Co., Ltd. (Cambridge, England). Antibodies against TOR, tubulin and GFP; secondary antibodies conjugated with horseradish peroxidase; and Lyso-Tracker Red, DAPI, Annexin V-FITC, WST-8 ([2-(2-methoxy-4-nitrophenyl)-3-(4-ni-trophenyl))-5-(2,4-disulfophenyl)-2H-tetrazolium, monosodium salt) were purchased from Beyotime Institute of Biotechnology (Nanjing, China).

### Cell culture

*S. litura* ovarian SL-1 cells were cultured in grace’-s insect cell culture medium (Gibco, Invitrogen, USA) supplemented with 10% heat-inactivated fetal bovine serum (HyClone, SV30087.02, Invitrogen, USA) at 27 °C. Exponentially growing SL-1 cells were plated at an appropriate density according to each experimental scale.

### Cell proliferation assay

Cell proliferation was determined using a WST-8 assay kit because it has little toxicity to cells and can be reduced to soluble formazan by dehydrogenase in mitochondria. Briefly, 1 × 10^4^ cells were seeded in a 96-well plate at 27 °C for 24 h. Subsequently, the cells were treated with different concentrations of AZA for 24 h. Cells incubated with 0.1% DMSO served as the control. After incubation with 10 μl WST-8 dye for 3 h, the absorbance was finally determined at 450 nm using a microplate reader (Bio-Rad 550, Japan). The inhibition rate was calculated as described in our previous study^27^.

### Transmission electron microscope (TEM) analysis

Cells at the density of 0.5 × 10^6^ −1 × 10^6^ cells/mL were incubated in 25 cm^2^ plates for 48 h. After treatment with AZA for different times (0, 12, 24, 48 or 72 h), the cells were fixed in 3% glutaraldehyde and 1.5% paraformaldehyde in 0.1 M PBS for 2 h. The samples were then washed with 0.1 M PBS, post-fixed in 1% osmium tetraoxide for 1 h, and embedded in Spur Resin according to the manufacturer’s instructions. From the fixed, the specimens were cut to 60 nm thickness and stained with 0.5% uranyl acetate and lead citrate. Finally, the autolysosomes and autophagosomes, as markers of autophagy^6^,^16^ were observed using a transmission electron microscope (Tecnai 12, FEI Co., the Netherlands). An important criterion for autolysosomes is the visible remnants of organelles and the undigested materials.

### Lyso-Tracker Red and DAPI staining analysis

Cells were cultured and treated with 2.5 μg/mL AZA as described above. Then, the cells were fixed with 4% paraformaldehyde for 15 min and washed with PBS three times. Finally, cells were stained with Lyso-Tracker Red for 2 h or DAPI for 15 min. The lysosomes and nucleus morphology changes were observed and photographed using a laser scanning confocal microscope (ZEISS, Germany).

### Annexin V apoptosis assay

Cells at the density of 2 × 10^5^/mL were incubated with AZA for different times and washed with PBS. After the addition of 195 μl binding buffer (10 mM HEPES, 140 mM NaCl, 2.5 mM CaCl_2_, pH 7.4), 5 μl FITC-labeled annexin V were added and incubated for 10 min, followed by incubation with 10 μl propidium iodide for 10 min. Then, apoptotic cells were analyzed using flow cytometry (BD FACSCalibur).

### Western blot analysis

After incubation with 2.5 μg/mL AZA for different times, the cells were collected and lysed with RIPA Buffer (1× PBS, 1% NP-40, 0.5% sodium deoxycholate, 0.1% SDS), to which inhibitors were added at time of use to the following final concentrations: 1 mM PMSF, 0.2 trypsin inhibitor U/mL aprotinin, 1 mM sodium orthovanadate). Protein concentrations were determined using a BCA kit (TIANGEN, China), and 30 μg protein was separated by 15% SDS-PAGE and transferred to a polyvinylidene difluoride (PVDF) membrane. After blocking, the membrane was probed with the appropriate antibodies. Finally, chemiluminescence detection was performed using the ECL detection system (Bio-RAD, USA).

### Vector construction

*S. litura Atg8* was amplified using cDNA prepared from the mRNA of SL-1 cells as a template for PCR. The plasmid pIEX-4 was used to express Atg8 fusion proteins. The fusion gene EGFP-Atg8 contains the EGFP gene without a stop codon and an *Atg8* gene with a stop codon was inserted into multi-cloning sites of pIEX-4. The primers used in this study are listed in Table 1.

Specific primers for FOXO and GFP genes were designed and PCR, RFP-GFP-LC3 fused gene amplified by PCR using pGTLV-RFP-GFP-LC3 lasmid as template. Using the vector of pIEx-4 and T4 ligase recombinant lasmid of pIEX4-RFP-GFP-LC3 and pIEX4- FOXO-GFP.

The various versions of Atg5, including the full-length, cleavage sites deletion and the cleaved fragments fused with the V5 tag at the N-terminal were amplified by PCR using total cDNA as template. Finally, the sequences were inserted into the pIEX4 vector (pIEX4-Atg5 and pIEX-4-V5-Atg5^△193–201^).

### Transient overexpression

SL-1 cells were seeded onto sterile confocal culture plate for 48 h. A transfection mixture was prepared by mixing plasmid pIEX-4-GFP-RFP-LC3, pIEX-4-GFP-Atg8, pIEX-4-V5-Atg5 and pIEX-4-V5-Atg5^△193–201^ (1 μg), enhancer 8 μl and Effectene transfection 15 μl for each well, according to the manufacturer’s instructions (QIAGEN, China). Then the medium was removed from the wells, the cells were washed three times with PBS, and the transfection mixture supplemented with fresh growth medium was gently added into the wells. The cells were then incubated with the mixture for 12 h at 27 °C. Finally, the cells were incubated with Grace’s insect medium supplemented with 10% FBS and observed using a laser scanning confocal microscope.

### Quantitative RT-PCR (qRT-PCR)

Levels of mRNA for *Atg8* were determined by RT-PCR. Total RNA from SL-1 cell lines was isolated using an RNA isolation system kit (Aidlab, China) and used to synthesize the first chain of cDNA with the cDNA Synthesis Kit (TaKaRa, Dalian, China) according to the manufacturer’s instructions.

qRT-PCR reactions were performed with three technical replicates on the BioRad CFX96^TM^ real-time PCR detection system using 200 ng of cDNA, 0.2 μM of primers and SYBR Premix Ex Taq (TaKaRa, China). The primers were designed with Primer 5.0 software (Table 1). The *tubulin* gene was used as a control. The mean value was calculated using three independent biological samples, and relative gene expression was analyzed according to the 2^−∆∆Ct^ method^62^.

### Statistical analysis

All results were from at least three separate experiments. The data were obtained by one-way analysis of variance using SPSS version13.0 (SPSS, Chicago, USA). In all statistical analyses, statistically significant differences were reported as *p < 0.05 or **p < 0.01. Data with values of p < 0.05 were generally accepted as statistically significant.

## Additional Information

**How to cite this article**: Shao, X. *et al*. Induction of Autophagy and Apoptosis via PI3K/AKT/TOR Pathways by Azadirachtin A in *Spodoptera litura* Cells. *Sci. Rep.* **6**, 35482; doi: 10.1038/srep35482 (2016).

## Figures and Tables

**Table 1:** Primers used in this study.

Primers	Primer sequence
**Primers for qRT-PCR**	
qATG8-F	AAGGCCAGGCTTGGAGAC
qATG8-R	GGCTGATGTTGGAGGAATG
qα-TUBULIN-F	CGCATTCATGGTTGATAACG
qα-TUBULIN-R	GGGCACCAAGTTAGTCTGGA
**EGFP-Atg8**	
EGFP(S*al*I)-F	GTTAGTCGACATGGTGAGCAAGGGCGAGGAG
EGFP-R	CTTCTTTATATTGGAATTTCATCTTGTACAGCTCGTCCATGC
ATG8-F	GCATGGACGAGCTGTACAAGATGAAATTCCAATATAAAGAAG
ATG8(HindIII) -R	GCCGAAGCTTTTAATATCCATAAACATTTTCATCAG
